# Sustainable flavor, healthy future: toward a green transition of the UPV in the food sector

**DOI:** 10.3389/fpsyg.2024.1445655

**Published:** 2025-01-08

**Authors:** Rebeca López López, María Belén Picó Sirvent, Débora Domingo-Calabuig, Javier Martínez-Monzó, Purificación García-Segovia

**Affiliations:** ^1^i-FOOD Team, IIA-FoodUPV, Universitat Politècnica de València, Valencia, Spain; ^2^COMAV, Universitat Politècnica de València, Valencia, Spain; ^3^Department of Architectural Design, Universitat Politècnica de València, Valencia, Spain

**Keywords:** sustainability, green transition, ecological, healthy eating, waste management, awareness

## Abstract

**Introduction:**

Due to the current climatic situation of the planet and the increase in concern for the environment, the Universitat Politècnica de València (UPV) aims to be a model for the university community in terms of the preservation of the ecosystem and prevention of the environmental impact caused by daily tasks; thus, aligning itself with the goals of the 2030 Agenda. For this reason, a project has been launched to carry out the green transformation of the UPV toward a university that prioritizes sustainability in all its areas.

**Methods:**

As part of this project, a survey was conducted using anonymous online questionnaires for the student population and employees. The study aimed to gauge the perception of sustainability and campus food supply and included items related to waste management and public awareness. A total of 800 students and 100 employees from the three UPV campuses (Vera, Alcoy, and Gandía) participated, ranging from 17 to 66 years old.

**Results:**

After the statistical analysis of the results, significant differences were identified in most of the questions of the different thematic blocks and, in some cases, in terms of gender and age group. In general, good knowledge about sustainability was observed, although participation in initiatives organized by the university was low in both population groups. On the other hand, as the age of the participants increased, a greater adoption of sustainable behaviors was observed, especially in buying and recycling habits. Regarding the food supply on university campuses, the need to improve it to promote healthier and more sustainable options is highlighted. This work investigates ways to improve the menu choices offered in university settings to promote healthier and more sustainable habits. Additionally, the study aims to identify potential obstacles within the university environment that may hinder these efforts, raise awareness, and encourage more environmentally friendly behaviors.

**Discussion:**

The proposed improvements include: (i) increasing the variety of plant-based options, (ii) sourcing food locally to reduce its carbon footprint, and (iii) implementing a waste management system that encourages recycling.

## 1 Introduction

The conception that people are in a new era of global environmental transformation is becoming increasingly consolidated, not only exerting an influence in most fields of science but also transcending the spheres of political decision-making and extending to the individual perception of citizens (Jiménez Herrero, 1999).

Due to the climate crisis, in July 2015, the final proposal for the SDGs (Sustainable Development and Goals), also known as the 2030 Agenda, was presented (Sanahuja and Tezanos Vázquez, 2017). It is comprised of 17 objectives; the plan presents an ambitious vision of global environmental sustainability, seeking to mitigate the risks associated with human-induced climate change. These goals, and in particular SDG 12, propose sustainable patterns of production and consumption, incorporating a significant environmental component that reflects the need to integrate sustainable practices in various areas of development to ensure the planet’s ecological viability (Gómez Lee, 2019). This SDG focuses on reducing waste generation and achieving sustainable use and management of natural resources (Sanahuja and Tezanos Vázquez, 2017).

On the other hand, social modernization has led to substantial transformations in food consumption patterns, as well as an increase in the production of food waste in domestic contexts. It is estimated that approximately every Spanish household wasted 65.5 kg of food and beverages [MAPA (Ministerio de Agricultura y Pesca), 2022]. According to 2013 FAO data and MAPA (Spanish Ministry of Agriculture, Fisheries and Food) in the report on food waste in households, foods non-processed foods, such as fruits, vegetables, and cereals, are the most wasted foods [Morata Verdugo et al., 2020; MAPA (Ministerio de Agricultura y Pesca), 2022].

There is now a growing awareness that it is in the hands of human beings to ensure that development must be sustainable and that this sustainability can be achieved through the adoption of changes in human behavior (de Rosselló, 2019). Associated with these social changes, concerns have arisen within society about acquiring healthier eating habits. These eating habits are understood as the set of customs through which individuals prepare, consume, and even select the foods they will eat (Sánchez Socarrás and Aguilar Martínez, 2015). In this context, food systems are increasingly associated with the environmental impact and economic and social effects that ultimately affect the planet’s sustainability (Prata Gaspar et al., 2023).

Political agents have developed social initiatives to promote sustainable consumption and reduce the impact of daily activities. Education can aid these initiatives (Deskin and Harvey, 2023). Specifically, universities can teach their students to generate a set of scientific and technological criteria that enables them to promote strategies and alternatives for development in different contexts of life (Botet et al., 2018). Therefore, universities can implement the relevant actions to address and respond to the challenges of the 2030 Agenda, serving as an experimental laboratory for sustainability (Gutiérrez and Pellegrini, 2022; Gutiérrez and Blanco, 2023). In fact, some actions aimed at university students concerning food sustainability have already been implemented. They are designed to expose students to initiatives that promote a more sustainable food system (Deskin and Harvey, 2023).

However, social perceptions of these issues are primarily based on subjective and socially integrated knowledge, so individuals perceive the concept of sustainability in various ways. Knowing the perceptions of staff and students at the university is essential to understanding the need to promote a critical perspective on sustainability and the healthy food system and where the misconceptions around the topic lie. For this reason, the current study was designed to analyze the perception of the university community of Universitat Politècnica de València (UPV, Spain) on sustainability, food, and waste management, within its three campuses. The main goal of this study was to investigate the perceptions of the university community regarding the issue of sustainability. A secondary objective was to evaluate their familiarity with the sustainability resources offered by the university and their understanding of the importance of adopting sustainable daily activities. With the project results, a strategy will be developed to raise awareness and educate them on their behavior to achieve healthier and more sustainable lifestyles.

## 2 Materials and methods

### 2.1 Questionnaire design

An anonymous survey was designed, consisting of 5 thematic blocks, considering open and multichoice questions. A general block included sociodemographic questions from the participants to find out their level of education, age, gender, and who they usually lived with, in addition to adding a question about whether they followed any particular diet. For the remaining sections, the survey focused on four key aspects: (i) the concept of sustainability, (ii) healthy and environmentally friendly eating with a particular focus on the food offered on campus, (iii) waste management and food waste, and (iv) environmental awareness, more focused on the issue of recycling. The survey questions were selected based on a literature review and adapted from similar studies on ecological transition in university settings (Cubero-Juánez et al., 2017). Initiatives carried out at the UPV, such as the studies promoted by CERAI (Centre for Rural Studies and International Agriculture) in the project Sustainable Campus Objective (Álvarez, 2021; Entrena-Durán et al., 2021) and specifically its survey on food supply in the UPV (Giner and Morales, 2021) were also referenced. The survey questionnaire was prepared online using SENSESBIT software (Sensesbit, 2024). It was available on the University’s platform, accessible to all UPV members (students and staff) through a link active 24 h a day. The survey lasted approximately 15–20 min. It was active for 10 days.

### 2.2 Statistics analysis

The results derived from the survey were expressed as means or frequencies according to the type of questions. Word association data was processed, grouping similar terms into categories. Word cloud representations of the frequency of these categories were obtained.

The statistical analysis to interpret and correlate the results obtained in the survey was carried out through the XLSTAT v.2023.1.4 program (Lumivero, 2024). The nonparametric variance was analyzed by comparing *k* samples with independent Kruskal-Wallis and Friedman data for samples with related data; results with a *p* < 0.05 are considered significant. The same statistical analysis was carried out to differentiate between age groups and genders for the two types of participants, students and staff.

## 3 Results

This survey was launched to the entire university community, obtaining participation from 900 individuals. This response rate corresponded to 2.5% of the student population and 2.3% of the staff, which included teaching, research, and administrative and service professionals. When analyzing the results, it must be considered that, due to the nature of the survey, the majority of participants were students, since they are more familiar with and active on the university’s website, where the survey in question was posted.

The staff survey’s sociodemographic results show that the respondents’ age ranges from 23 to 66, with the highest participation from people over 51 (33.4%). In the students’ survey, the ages ranged from 17 to 61 years, with the highest percentage of participation among respondents between 21 and 23 years of age (32.4%).

Regarding gender, female participation is predominant among staff: 61.8 and 38.2% of women and men participated, respectively. Different studies have reported this trend, such as those by Becker (2022), Green (1996), and Becker and Glauser (2018), which show a higher likelihood of responses by women in online surveys. Similarly, in an analysis carried out by Porter and Umbach (2006), it was found that male participants showed a much lower response rate than women, giving as a possible explanation for this gender gap how men and women value actions and make decisions in the online environment (Smith, 2008; Wu et al., 2022). However, among students, there was a more balanced gender distribution, with 48.9% women and 50.3% men. A minority group (0.8%) was added that was classified as ‘I do not identify with binary gender.’

Most of the staff respondents (45.6%) lived with a partner or spouse and children. In comparison, a higher percentage of students lived with their parents or close relatives (48.4%). Concerning the education level, 86.8% of the staff surveyed had a higher level of studies, master’s or doctorate; however, in the student survey, only 11.13% marked this option, with the bachelor’s degree being the predominant option. When asked whether or not they followed a particular diet, in both cases, most respondents (70.6 and 62.5%) indicated that they did not follow any diet but were worried about it.

In the following section, the data obtained will be presented and separated by thematic blocks, including the results of the two types of surveys (students and staff).

### 3.1 Block 1: sustainability concept

Table 1 shows the results from the first block, corresponding to the degree of knowledge respondents held about sustainability and the various initiatives and services related to the environment within the UPV.

**Table 1:** Block 1: sustainability concept.

Question	Answer	Staff %	*p*-value	Students %	*p*-value
In general, how do you consider yourself to be informed on the subject of the environment?	Nothing	0.00	<0.0001	0.27^a^	<0.0001
	Next to nothing	0.00		2.28^a^	
	Bit	5.88^a^		14.21^b^	
	Something	50.00^b^		60.32^d^	
	A lot	44.12^b^		22.92^c^	
Do you know the term sustainability? From the following six concepts, choose the three that you most associate with sustainability	Reuse of packaging	19.72^bc^	<0.0001	19.17^c^	<0.0001
	Organic farming	12.68^ab^		13.09^b^	
	Electric Transport	7.51^a^		9.45^a^	
	Renewable energy	26.76^c^		28.84^d^	
	Biodegradable materials	18.31^bc^		21.14^c^	
	Separate waste collection	15.02^ab^		8.32^a^	
Have you participated in or are you aware of any university initiatives on the environment?	Yes, I know and have participated	39.71^a^	0.054	11.93^a^	<0.0001
	Yes, I know, but I have not participated	38.24^a^		43.30^b^	
	No, I do not know, and I have not participated	22.06^a^		44.77^b^	
Is there a unit/office in your institution dedicated exclusively to sustainability issues?	Yes	60.29^b^	<0.0001	33.24^b^	<0.0001
	No	14.71^a^		4.02^a^	
	I do not know	25.00^a^		62.73^c^	
Through what means should your university communicate its sustainability initiatives?	Web page	30.64^b^	0.000	23.96^b^	<0.0001
	Reports/Mail	24.28^ab^		25.33^b^	
	Social Media	27.17^b^		34.41^c^	
	Congresses/conferences/seminars	17.92^a^		16.30^a^	
Do you consider environmental education necessary in the university’s academic programs*	Yes	86.76^b^	<0.0001	–	–
	No	1.47^a^		–	
	I do not have an opinion	11.76^a^		–	
Do you think the university has an environmental impact on its daily life?	Yes	92.65^b^	<0.0001	76.14^c^	<0.0001
	No	0.00		3.62^a^	
	I have not thought about it	7.35^a^		20.24^d^	
What do you think are the barriers to making the university sustainable? Choose 3 of the following proposals:	Lack of motivation/awareness of students	18.18^ab^	0.004	25.07^d^	<0.0001
	Economic or financial constraints	18.72^ab^		16.62^bc^	
	Pedagogical limitations due to lack of curricular adaptation	9.63^a^		10.12^a^	
	Lack of communication	13.37^ab^		15.14^b^	
	Logistical and structural difficulties of the university	18.72^ab^		14.71^b^	
	Lack of resources/alternatives	21.39^b^		18.34^c^	

*p-value < 0.05 indicates statistically significant differences in responses between. The same letters in columns indicate homogeneous groups according to Dunn’s procedure.*

^*^For the students, two other questions refer to the teaching plan.

In the first question on the degree of knowledge about environmental issues, significant differences (*p* < 0.0001) were observed among UPV staff, particularly between the responses that correspond to little to no knowledge (categories of “nothing,” and “next to nothing”) and the other responses (“a bit,” “something,” and “a lot”). The ‘little to no options were the least chosen, indicating that the participants generally possess some level of knowledge about environmental issues. Furthermore, significant differences were noted across age groups, with individuals over 51 as the most knowledgeable on this topic, because they chose the option “A lot” with the highest frequency (14.7%).

In the student survey, the overall *p*-value was also significant (*p* < 0.0001) with results similar to those of staff; however, no significant differences were found when analyzing the data by gender and age ranges.

The next question dealt with the term *sustainability*, in which the participants had to choose three of the six concepts that they related to sustainability; in both cases, significant differences appeared (*p* < 0.0001); the most selected terms were renewable energies, packaging reuse, and biodegradable materials. This indicates that the surveyed population associates sustainability with second life and reusing materials. Significant demographic differences were observed only among age groups between >23 and < 19 to 21 with a *p* = 0.001 for the selective waste collection response in the student survey. No significant differences were found when analyzing the data by gender.

The following question was about the participation or knowledge of the environmental initiatives offered at the university. The results demonstrated significant differences in the student survey (*p* < 0.0001) between the response “I know and have participated” and the other responses, with this option being the least selected by students (only 11.9%). Conversely, this option was the most selected by staff (39.7%). This indicates that the student community needs to be more closely engaged with the environmental initiatives led at the university, as low participation poses a limitation to the university’s efforts in transitioning to an eco-friendlier system.

In both surveys, the respondents were asked if they knew of a university service dedicated to sustainability issues. The results showed significant differences in surveyed groups (*p* < 0.0001). The answer was satisfactory for staff (60.3%) but displayed a lack of knowledge among the student community (33.2%). This may be due to a lack of interest on the part of this group or because the means of communication are not appropriate. For this reason, the following question was asked.

The following question addressed which medium would be the most effective for publicizing information about events related to sustainability at the University; only in the case of students were there significant differences in age and gender. The mediums most voted for by women were social networks (53.08%), while men selected other options, such as the University’s website (56.1%). Therefore, it would be interesting to use the most voted communication channels as a means of communicating this type of initiative or activity.

**Figure 1:**
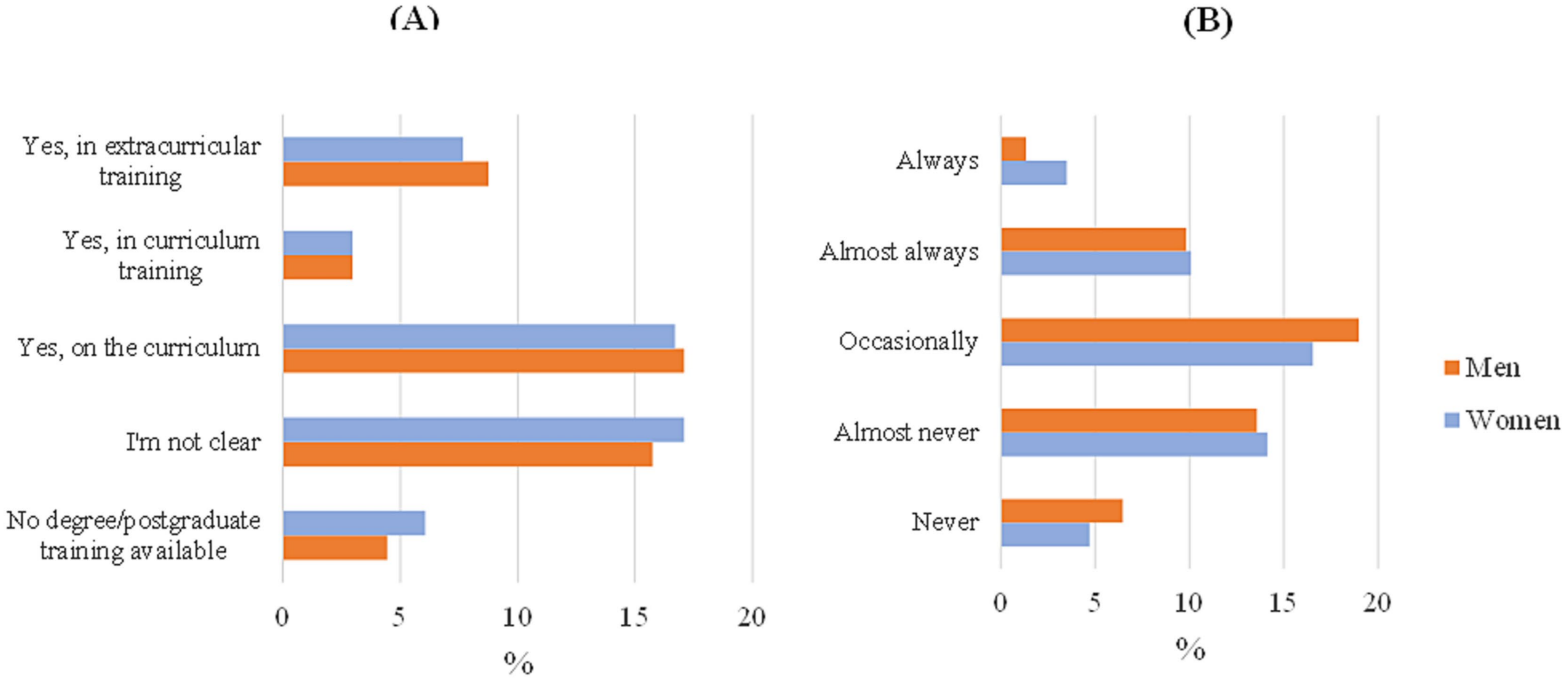
Answer students by gender. **(A)** Do you consider that the topic of sustainability is incorporated into your training? **(B)** Do teachers in class emphasize sustainability issues? Do they include sustainability issues?

The next question, “Do you consider environmental education necessary in the university’s academic program?” was only presented in the survey for staff; students were asked two other questions that were more focused on their teaching plan. In the first case, significant differences were found between respondents in general, since there was a very high difference between the answer ‘yes’ (86.8%) and the rest of the options, so, in general, there is awareness of the importance of introducing these topics in educational programs. The questions that were asked to the students instead were the following: “Do you consider that the topic of sustainability is incorporated into your training?” and “Moreover, “Do teachers in class emphasize sustainability issues? Do they include sustainability issues?” For the first question, significant differences appeared especially between age groups; students >23 indicated with a higher percentage the option “On the curricular “as well as the age group from 21 to 23, but the rest of the age groups were largely unclear. Similarly, significant differences were found in the answers to the second question, as most students answered “Occasionally.” These differences can be seen in Figure 1.

In the second-to-last question, “Do you think the university has an environmental impact on its daily life?” There were also significant differences (*p <* 0.0001) between the answers in both surveys, bein the staff more aware of this impact than the students (answering “yes” 92.7% vs. 76.1%).

The answers of both surveys for the last question of block 1, “What do you think are the barriers to making the university sustainable?” also had significant differences (*p =* 0.004 and *p* < 0.0001) for staff and students, respectively. The most voted for and, therefore, the issue that was considered the main obstacle to the university’s green transition was the lack of resources and alternatives for staff and lack of motivation or awareness on the part of the students. In addition, significant differences were also found between the different age and gender groups in both surveys. In the staff survey, there were significant differences (*p* = 0.004) between men and women for the option “Economic limits” and a *p* = 0.019 in terms of age between >51 and < 28, the latter being the group that voted the most for the “Lack of communication” option. In the student survey, age differences were found between the >23 and < 19 groups (*p* = 0.02) for the “Lack of communication” option. For the option “No resources/alternatives in their menus,” significant differences appeared between the three genders, as well as the <19 and the 21 to 23 age groups.

**Figure 2:**
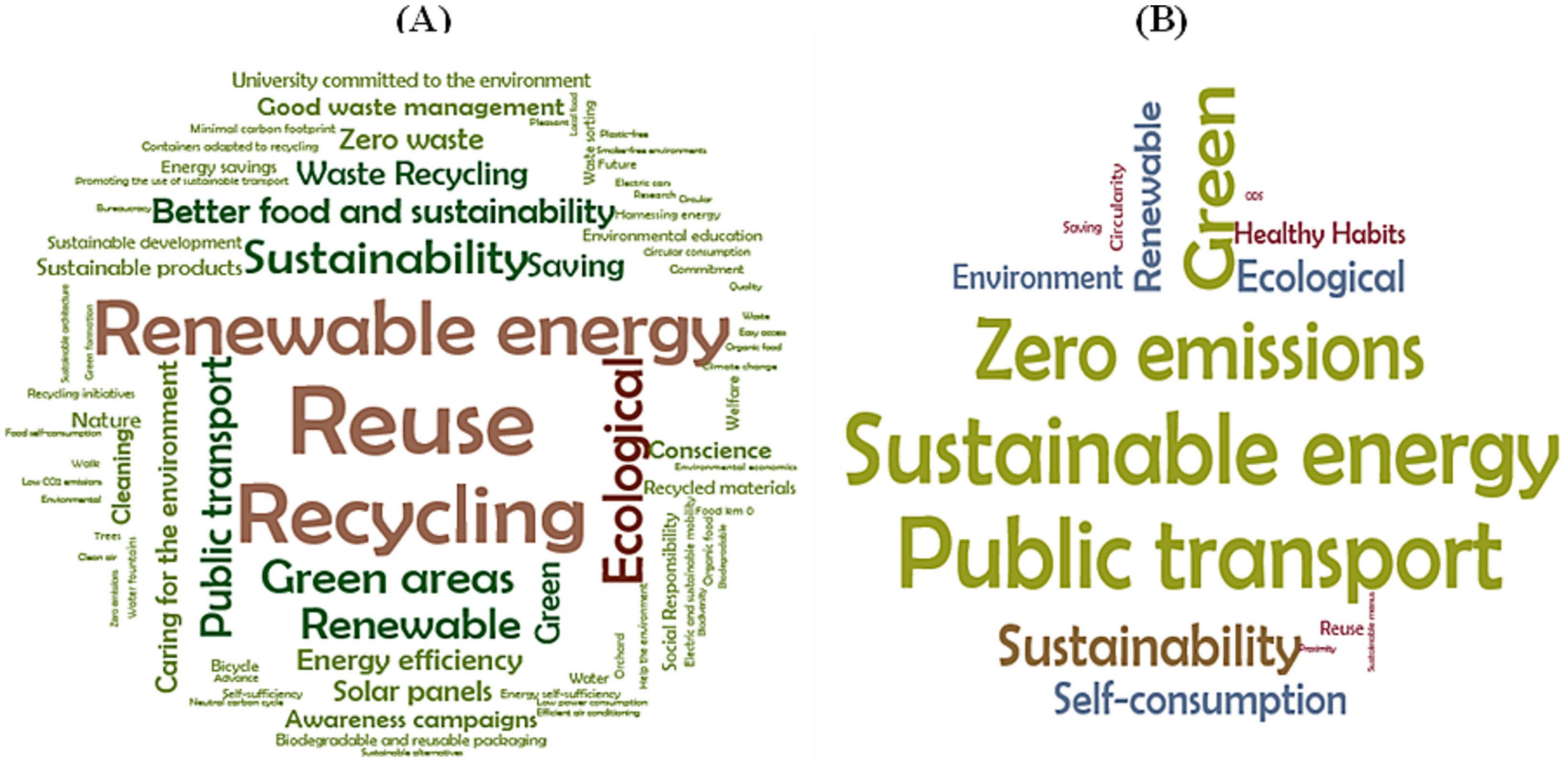
Word cloud of elicitation frequency for concepts about green, eco or sustainable campus: **(A)** students, **(B)** staff.

Within this block, an open-ended question was included, in which the survey participants had to write three concepts related to green campus, eco campus, or sustainable university. In this case, a compilation of all the words was made, which were then grouped into similar concepts, noting their frequency to finally form a word cloud, as seen in Figure 2.

As shown in Figure 2A, for students, “reuse,” “recycling,” and “renewable energy” were the most repeatedly used words to describe a green campus or sustainable university. To a lesser extent, but with high frequency, they also associated university sustainability with “public transport,” “green areas,” “ecology,” or “more sustainable food.” In Figure 1B, the most prominent concepts elicited by staff were “zero emissions,” “sustainable energy,” and “public and green transport,” and the word “sustainability” also appeared less frequently.

### 3.2 Block 2: healthy and environmentally friendly eating

Table 2 presents the results from the second block of questions related to food, specifically that offered on the UPV campus. Except for the question, “Are seasonal and local products actively promoted on campus?” for staff (*p* = 0.659), significant differences were observed in the responses to each question in both surveyed groups.

**Table 2:** Block 2: healthy and environmentally friendly eating.

Question	Answer	Staff %	*p*-value	Students %	*p*-value
How would you consider your diet?	Traditional (local)	23.53^b^	<0.0001	37^b^	<0.0001
	Vegetarian	1.47^a^		3.75^a^	
	Mediterranean diet (Healthy)	72.06^c^		47.72^c^	
	Vegan	0.00		1.47^a^	
	Flexitarian	1.47^a^		4.83^a^	
	Detox	0.00		0.27^a^	
	Hypocaloric/hypercaloric	1.47^a^		4.96^a^	
Do you think adequate nutrition should be considered a right to be promoted by the university?	Yes	91.18^b^	<0.0001	83.11^c^	<0.0001
	No	4.41^a^		4.96^a^	
	I do not have an opinion	4.41^a^		11.93^b^	
Do you think there is a need for any change in the UPV’s food system?	Yes	61.76^b^	<0.0001	46.11^c^	<0.0001
	No	11.76^a^		17.16^a^	
	I do not have an opinion	26.47^a^		36.73^b^	
How would you improve it? Choose 3 of the following proposals:	Menus with more eco-friendly products	13.30^a^	<0.0001	16.51^b^	<0.0001
	More Vegan/Vegetarian Menu Options	9.57^a^		11.51^a^	
	Highest number of km0 product	23.40^b^		19.55^c^	
	Less caloric menus	17.02^ab^		11.27^a^	
	Greater variety of dishes	19.15^b^		22.88^d^	
	More fruit on offer	17.55^ab^		18.27^bc^	
How often do you consume km0 or local products?	I never consume	0.00	0.001	3.22^a^	<0.0001
	Almost never	10.29^a^		15.82^b^	
	Occasional use	38.24^b^		43.16^d^	
	Every month	32.35^b^		24.8^c^	
	I consume every day	19.12^ab^		13.00^b^	
Do you think there is enough choice of menu dishes according to the Mediterranean diet/specific diets (vegan, celiac disease, lactose-free, etc.) in the UPV cafeterias?	Yes	26.47^a^	<0.0001	34.32^a^	<0.0001
	No	73.53^b^		65.68^b^	
Do you think UPV’s cafeterias’ menus should incorporate organic, seasonal, or local products to make them more sustainable?	Yes	83.82^b^	<0.0001	80.29^c^	<0.0001
	No	1.47^a^		4.96^a^	
	I am indifferent	14.71^a^		14.75^b^	
Would you be willing to pay more for it?	Yes	66.18^b^	0.000	58.04^b^	<0.0001
	No	33.82^a^		41.96^a^	
Are seasonal and local products actively promoted on campus?	Yes	29.41^a^	0.659	30.29^a^	<0.0001
	No	33.82^a^		26.01^a^	
	I have rarely seen any initiative promoting it	36.76^a^		43.70^b^	
What do you think the food offered in vending machines is like?	Not at all healthy	25.00^b^	<0.0001	19.71^b^	<0.0001
	Unhealthy	50.00^c^		43.30^c^	
	Healthy	0.00		2.41^a^	
	Very caloric	1.47^a^		4.42^a^	
	Not very varied	2.49^a^		5.50^a^	
	To eat occasionally	20.59^ab^		24.66^b^	
What are the three most important characteristics of a sustainable food product?	Use renewable energies during the production process	7.96^ab^	<0.0001	11.38^b^	<0.0001
	Composite packaging with biodegradable materials	15.42^bc^		17.59^cd^	
	Animal welfare information	3.48^a^		7.85^a^	
	Reduce greenhouse gas emissions during production	17.91^c^		19.22^d^	
	Contribute to the development of the local economy	23.88^c^		15.83^c^	
	Limit waste during production	22.89^c^		19.05^d^	
	Promoting organic farming	8.46^ab^		9.08^ab^	

*p-value < 0.05 indicates statistically significant differences in responses between. The same letters in columns indicate homogeneous groups according to Dunn’s procedure.*

In the first question regarding the type of diet followed by participants, the “Mediterranean diet (healthy) was the most selected option by both staff (72.06%) and students (47.7%). Significant differences (*p* < 0.05) were observed in the student survey based on gender. Specifically, the Mediterranean diet was predominant among women (26%), while the traditional diet was more common among men (20.7%).

For the second question, both groups, staff and students, mostly agree with the fact that adequate food should be considered a right (91.2 and 83.1%). The data indicated significant gender differences in both surveys. Nonetheless, the predominant response in both cases was that food should be regarded as a right promoted by the university. Both surveyed groups also think there is a need for a change in the food system of the UPV (61.8 and 46.1%) and to the question, “How would you improve it?” 23.4% of the staff suggested adding more Zero km food (products that are sold and consumed locally, and therefore travel zero kilometers from their production site to their consumers) to the dishes, while 22.9% of students recommended introducing a greater variety of dishes on the menus. Significant differences (*p* < 0.0001) were observed in both surveys concerning age and gender for the student responses. Men suggested the addition of more variety of dishes and more Zero km food (56.2 and 58.3% respectively), while women opted for the inclusion of more vegan dishes (63.1%).

Both groups indicate occasional consumption of Zero km and local food (38.2 and 46.2% for staff and students respectively). Significant differences were identified in both surveys (*p* = 0.001 and *p* < 0.0001), especially in women. Also, most of the respondents answered “No” to the question “Do you think there is enough choice of dishes on the menus according to the Mediterranean diet/specific diets (vegan, celiac disease, lactose-free, etc.) in the UPV cafeterias?” (73.5 and 65.7%). Consistently, when asked if the menus should incorporate more organic, seasonal, or local products, approximately 80% of both groups responded affirmatively, and most of them agreed with the idea of paying more for dishes with this type of product (66.2% of staff and 58% of students responded affirmatively).

In the question of the promotion of seasonal or local products within the campus, significant differences were found only in the student survey. The results indicated that both men and women were generally unaware of any initiatives for the promotion of these products. However, age-related differences were observed, as only the younger participants were familiar with the university’s efforts in this area.

**Figure 3:**
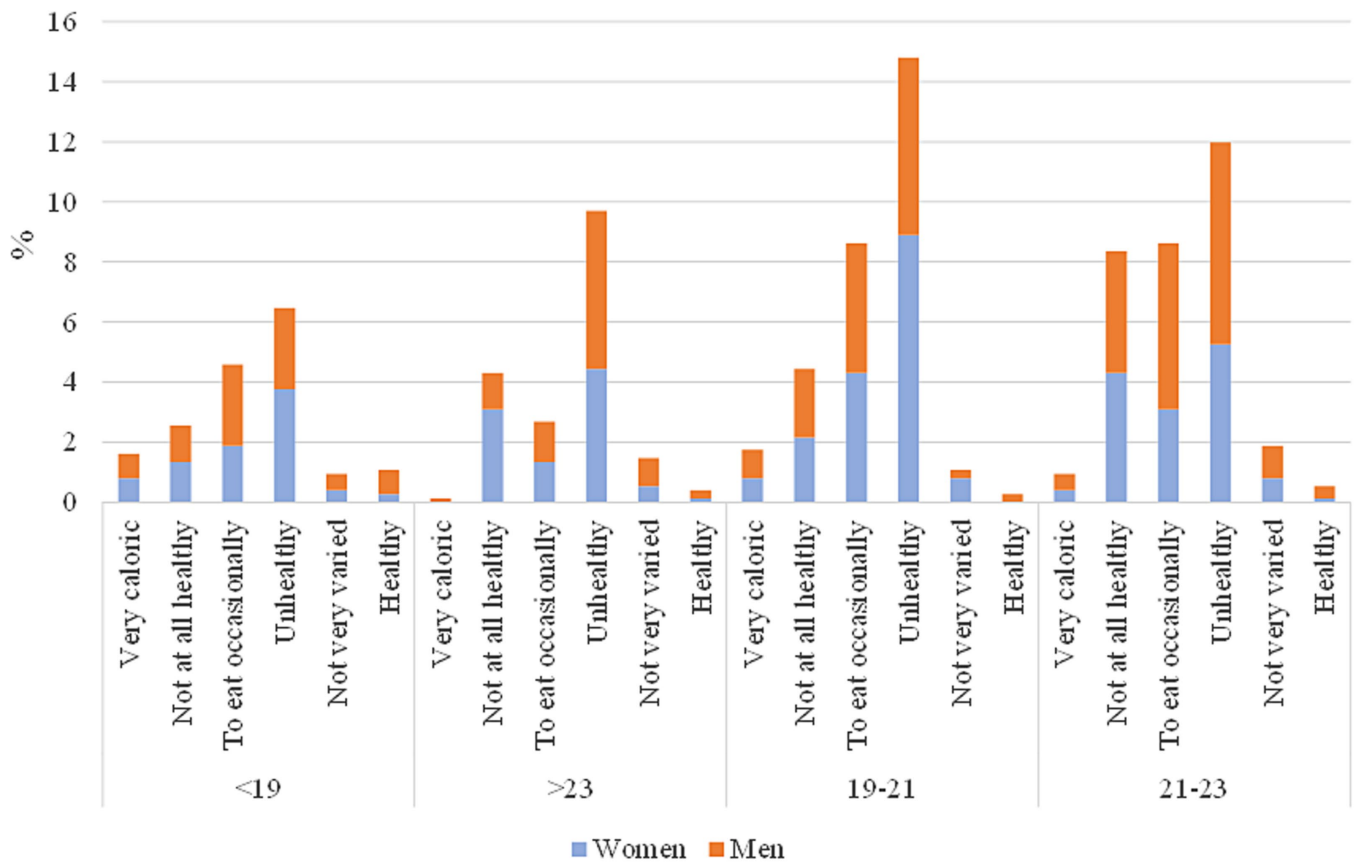
Student answer of the question: What do you think the food offered in vending machines is like?

Regarding the last question about the food from vending machines across the campus and their quality, significant differences were observed only among different age groups of students. As you can see in Figure 3, respondents aged 19–23 tended to view such food as unhealthy, preferring to consume it occasionally, unlike other age groups.

**Figure 4:**
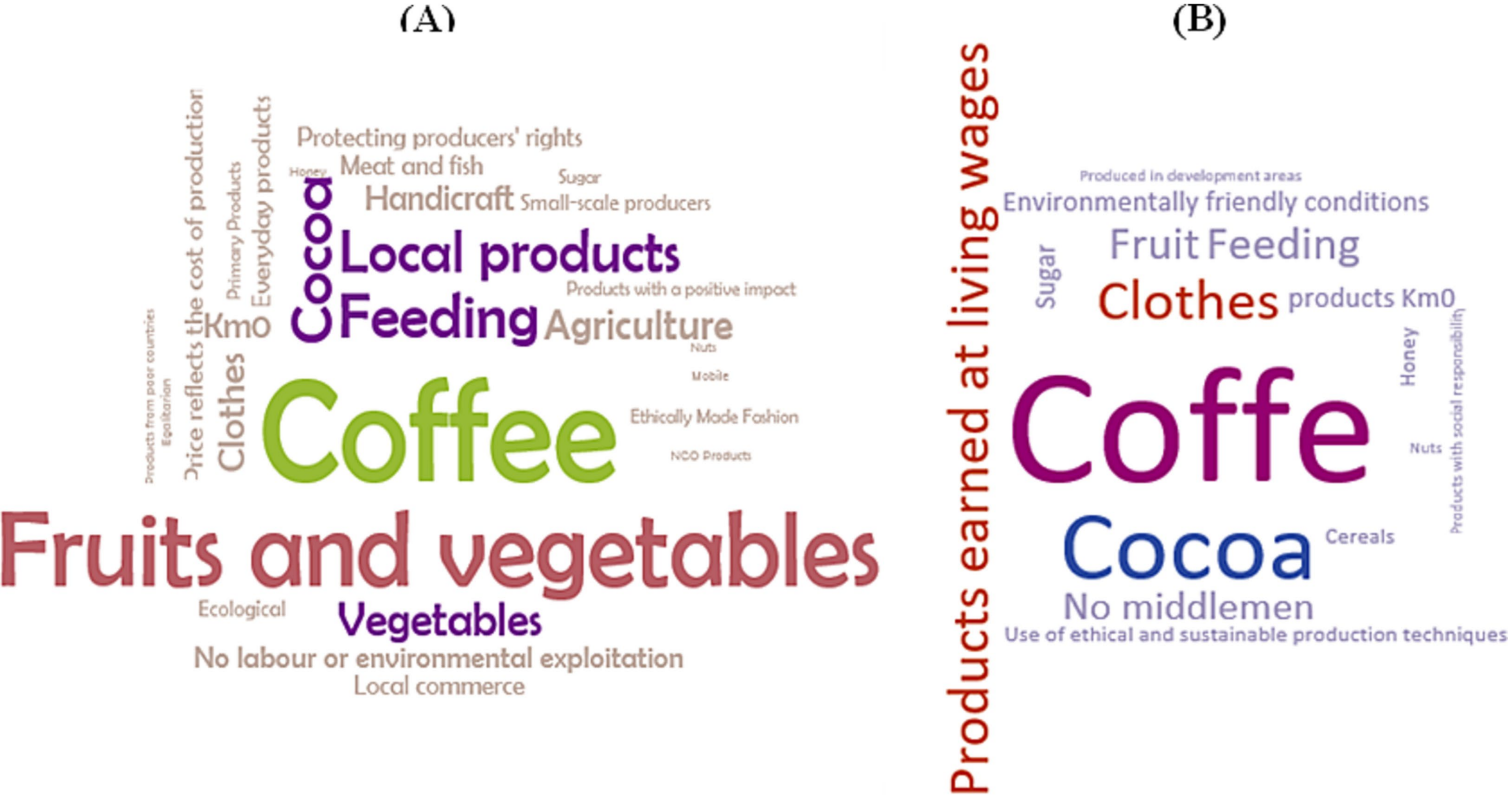
Word cloud of elicitation frequency for products related to fair trade **(A)** students, **(B)** staff.

In response to the question, “What do you think are the three most important characteristics of a sustainable food product?” significant differences were observed in both surveys. Staff prioritized the development of the local economy more highly (23.9%), with significant age-related differences (*p* = 0.005). Conversely, students considered reducing gas emissions during processing to be more critical (19.2%). When asked about fair trade, most students were unfamiliar with the term, unlike the staff (Figure 4).

An open question was included, in which participants were asked to write down the products they associated with fair trade. The responses were used to create a word cloud, as seen below:

As can be seen in Figure 4A, the most mentioned words associated with fair trade were “coffee,” “fruits,” and “vegetables,” next to “cocoa,” “local products,” “feeding,” and “agriculture” and “clothes” to a lesser extent. In Figure 4B, “coffee” was the most prominent, followed by “cocoa” and “clothes” and, to a lesser extent, “products earned at living wages.”

### 3.3 Block 3: collection of waste and food waste

The results of this block are gathered in Table 3. In the first question, respondents were asked about the adequacy of waste management practices at the university. The results indicated significant differences in general, obtaining a similar percentage of positive considerations of waste management within the university in both cases: 83.8% in the staff survey and 82.2% in the student survey. When analyzing the age groups of the students, significant differences were found between those <19 and those over 23. The staff’s questionnaire did not show substantial differences in terms of gender or age.

**Table 3:** Block 3: collection of waste and food waste.

Question	Answer	Staff %	*p*-value	Students %	*p*-value
Do you think that within the university, there is good waste management (organic, paper, plastic, etc.)?	Yes	83.82^b^	<0.0001	83.24^b^	<0.0001
	No	16.18^a^		16.76^a^	
Are awareness campaigns, environmental education, and recycling promotion carried out?	Yes	45.59^b^	0.009	35.25^b^	<0.0001
	No	20.59^a^		13.54^a^	
	I do not know	33.82^ab^		51.21^c^	
How often do you recycle?	Never	0.00	<0.0001	1.34^a^	<0.0001
	Almost never	2.94^a^		6.70^a^	
	When I remember	1.47^a^		12.73^b^	
	Usually	39.71^b^		44.24^d^	
	Always	55.88^b^		34.99^c^	
We are all concerned about food waste, but are you aware of the kg of waste you generate per year? Indicate the option of the amount of kg you think you generate in a year	60 kg	19.12^a^	0.035	14.21^a^	<0.0001
	75 kg	16.18^a^		16.62^b^	
	90 kg	29.41^a^		40.75^c^	
	121 kg	35.29^a^		28.42^a^	
Do you know the concept of circular economy?	Yes	85.29^b^	<0.0001	67.69^b^	<0.0001
	No	2.94^a^		15.68^a^	
	I do not have an opinion	11.76^a^		16.62^a^	
How often do you use Too Good to Go or similar services?	Never	58.82^b^	<0.0001	45.44^d^	<0.0001
	Sometimes	20.59^a^		30.03^c^	
	Occasionally	11.76^a^		18.10^b^	
	Regularly	8.82^a^		5.76^a^	
	Every day	0.00		0.67^a^	
To reduce the environmental impact of waste from the agri-food sector, to what extent do you think each of the following activities is effective? Not important Unimportant Somewhat important Important Very important	Reduction in packaging weight	0.00	0.002	6.70^a^	<0.0001
		13.24^a^		21.05^c^	
		30.88^ab^		32.17^d^	
		38.24^b^		25.60^c^	
		17.65^a^		14.48^b^	
	Reuse of food packaging	0.00	<0.0001	0.94^a^	<0.0001
		1.47^a^		3.49^a^	
		5.88^a^		10.05^b^	
		38.24^b^		33.38^c^	
		54.41^b^		52.14^d^	
	Recycling waste	0.00	<0.0001	1.14^a^	<0.0001
		0.00		1.14^a^	
		4.41^a^		5.36^a^	
		25^b^		24.93^b^	
		70.59^c^		67.43^c^	
	Reuse of food products	0.00	<0.0001	2.28^a^	<0.0001
		5.88^a^		6.17^a^	
		14.71^ab^		21.85^b^	
		33.82^bc^		37.13^c^	
		45.59^c^		32.57^c^	
	Recovery of packaging and food products for energy production	0.00	<0.0001	1.07^a^	<0.0001
		1.47^a^		3.22^a^	
		5.88^a^		10.72^b^	
		39.71^b^		25.74^c^	
		70.59^b^		59.25^d^	

p-value < 0.05 indicates statistically significant differences in responses between. The same letters indicate homogeneous groups according to Dunn’s procedure.

In the question, “Are awareness campaigns, environmental education, and recycling promotion carried out?” Significant differences were found in the two surveys in general, staff were more aware than students of these campaigns (45% vs. 35.3%).

Consistently, staff recycles more frequently than students (answering “Always” or “Usually” 95.6% vs. 79.2%, *p* < 0.0001).

**Figure 5:**
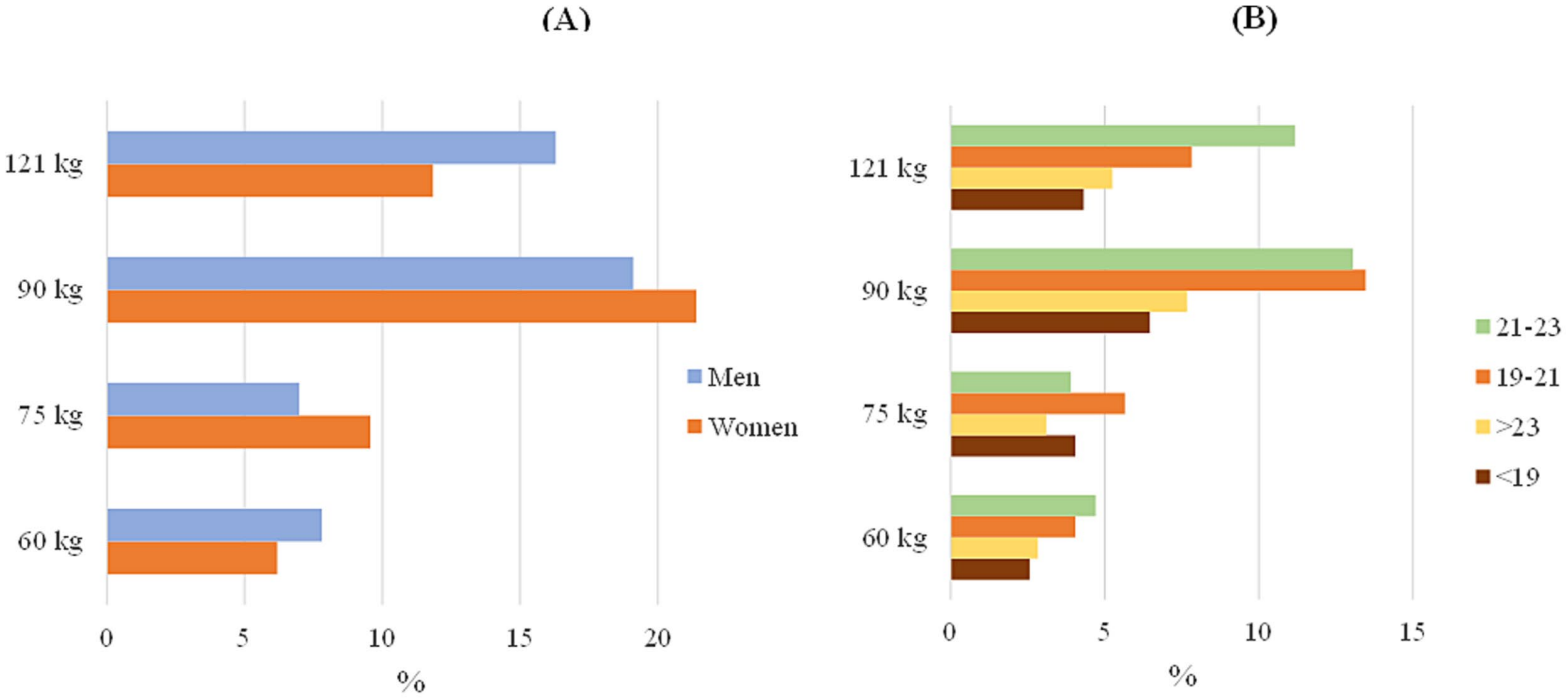
Student answer to the question: are you aware of the kg of waste you generate per year? **(A)** By gender; **(B)** by age.

The next question was about the annual quantity of waste generated by an individual, in which each respondent indicated the amount in kg of waste that they thought was generated. Significant differences were obtained for staff only at the general level (*p* = 0.035) and at the age and gender level for students as you can see in Figure 5. In the overall calculation of the student and staff surveys, the maximum percentage of answers was obtained in the options of 90 kg and 121 kg, respectively, so staff thinks that they produce more waste (Table 3).

The same trend was observed in the question regarding their knowledge of the concept of circular economy, in which 85.3% of the staff respondents and 67.7% of students answered that they were aware of it. Because most of the participants knew this concept, we wanted to know if they put this circular economy into practice through services that are within their reach.

The results obtained for the question on the frequency with which they use services such as Too Good to Go (a mobile app in which t excess food or products from restaurants and stores that have not been sold on that day but are still fit to consume, are sold to users at a lower price) shows that, in general, respondents do not use this type of service. In addition, it was observed that male staff use it less regularly.

Finally, when answering the question “To reduce the environmental impact derived from waste from the agri-food sector, to what extent do you think each of the following activities is effective?” respondents had to classify each impact according to its importance, from “not important” to “very important.” For this reason, a statistical analysis was conducted individually for each activity. In all cases, significant differences were detected. In general, both staff and students considered that Recycling waste, Recovery of packaging and food products for energy production, and Reuse of food packaging are more effective than the Reuse of food products and Reduction in packaging weight to reduce the environmental impact of waste for the agri-food sector. Also, a statistical analysis was made using gender and age ranges. The first activity that respondents were asked to evaluate was the reduction in packaging weight. There were differences in gender in the survey of staff, where women see it more important than men, and in the survey of students, where respondents aged 19–23 gave greater importance to this activity. The second activity was the reuse of food packaging and only differences between genders (*p* = 0.034) in the student survey. The same happened to the effects of waste recycling. In the second-to-last activity (Reuse of food products), significant differences were observed between genders in the student survey only, where women attached more importance to these activities than the other genders surveyed. The same thing happened in this question’s last activity (recovery of packaging and food products for energy production).

### 3.4 Block 4: awareness

**Figure 6:**
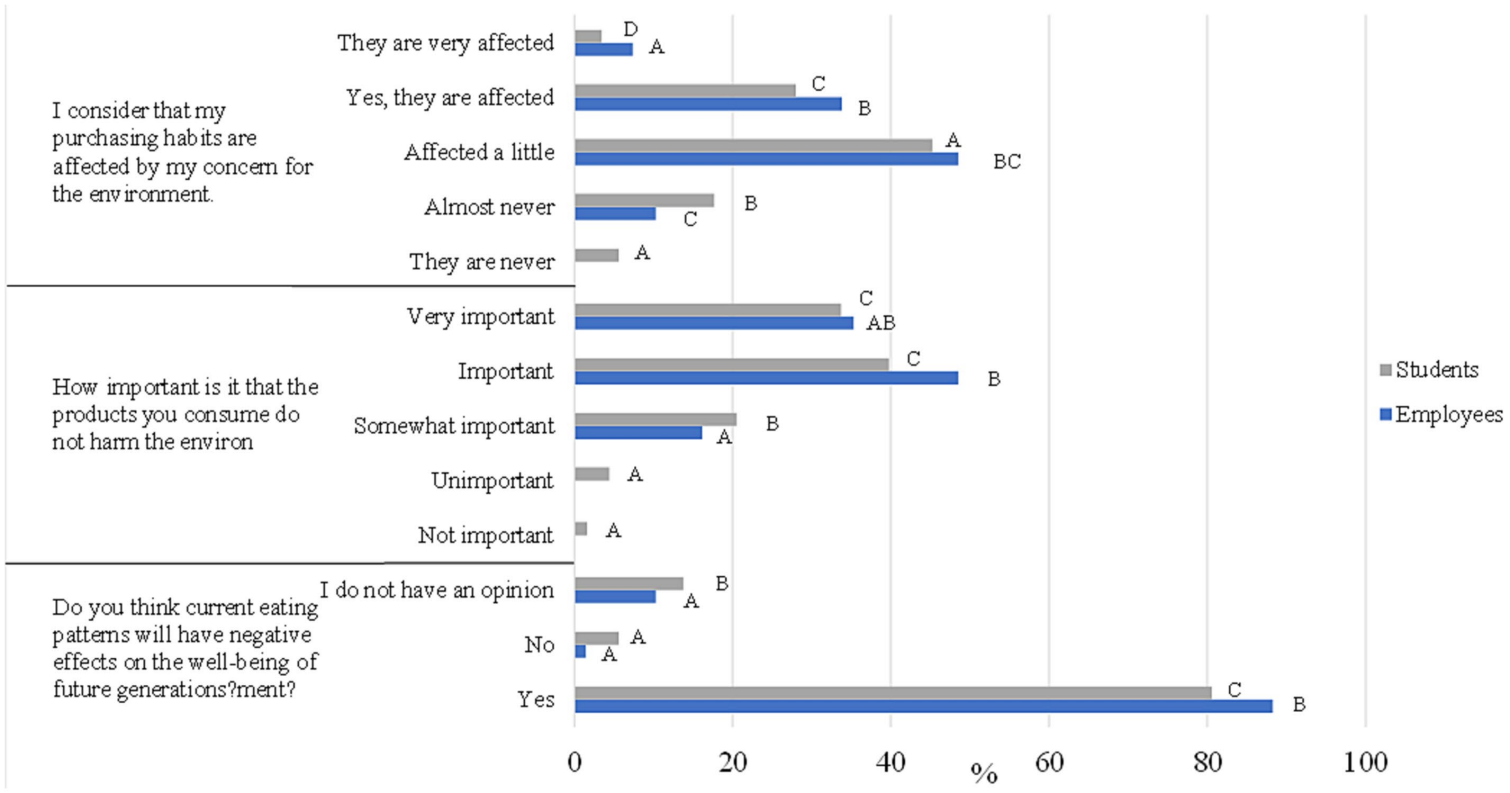
Student and employee answers to the first three questions of block 4.

**Figure 7:**
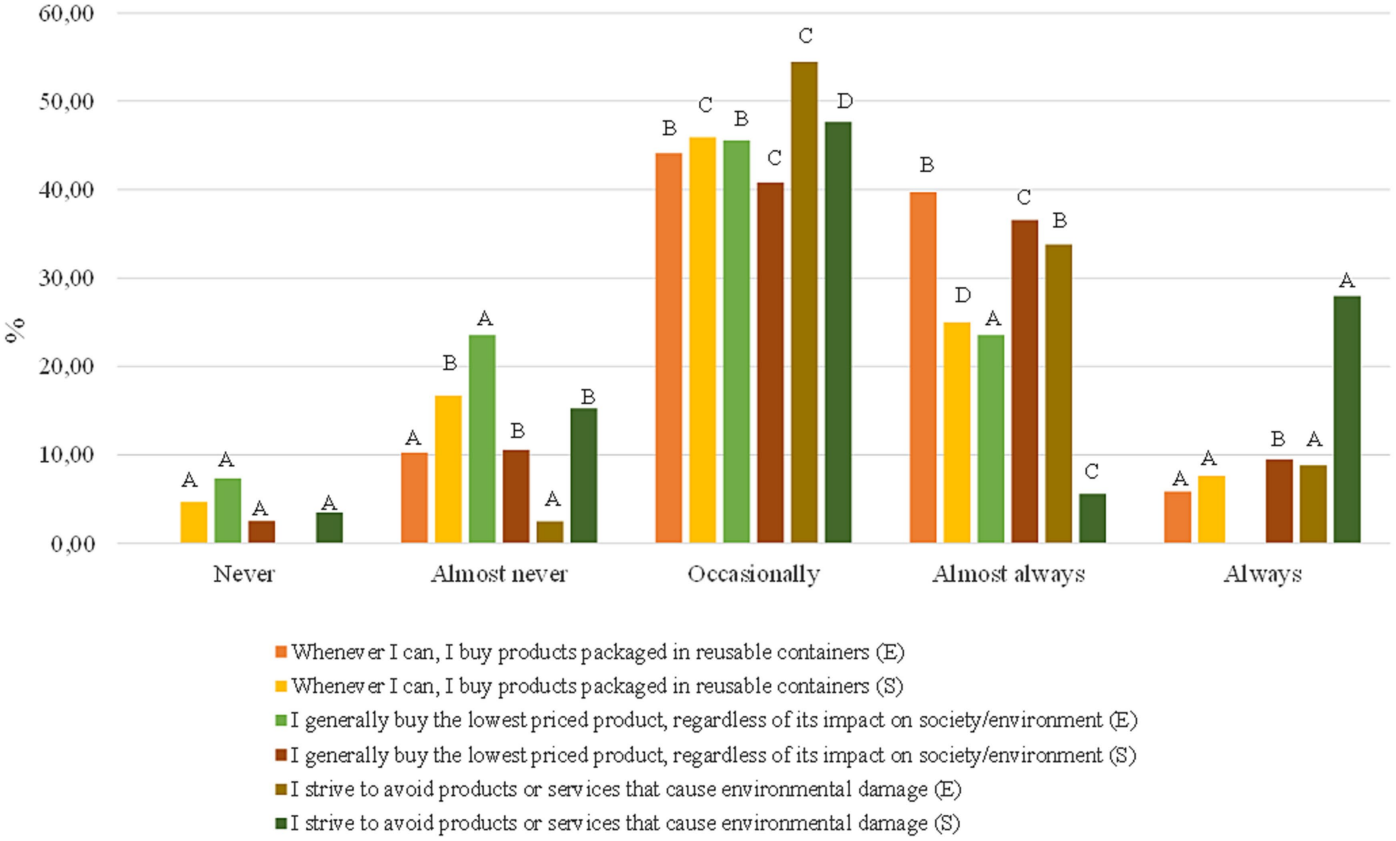
Student and employee answers to the last three questions of block 4.

This last block focuses on how our environmental awareness affects daily practices; in Figure 6, you can see the first 3 questions of the block, and in Figure 7, the last 3 questions (For more information, see Table 4).

**Table 4:** Block 4: awareness.

Question	Answer	Staff %	*p*-value	Students %	*p*-value
Do you think current eating patterns will have negative effects on the well-being of future generations?	Yes	88.24^b^	<0.0001	80.59^c^	<0.0001
	No	1.47^a^		5.62^a^	
	I do not have an opinion	10.29^a^		13.79^b^	
How important is it that the products you consume do not harm the environment?	Not important	0.00	0.000	1.61^a^	<0.0001
	Unimportant	0.00		4.42^a^	
	Somewhat important	16.18^a^		20.48^b^	
	Important	48.53^b^		39.76^c^	
	Very important	35.29^ab^		33.73^c^	
I consider that my purchasing habits are affected by my concern for the environment.	They are never	0.00	<0.0001	5.62^a^	<0.0001
	Almost never	10.29^c^		17.67^b^	
	Affected a little	48.53^bc^		45.25^a^	
	Yes, they are affected	33.82^b^		27.98^c^	
	They are very affected	7.35^a^		3.48^d^	
Whenever I can, I buy products packaged in reusable containers.	Never	0.00	<0.0001	4.69^a^	<0.0001
	Almost never	10.29^a^		16.73^b^	
	Occasionally	44.12^b^		45.92^c^	
	Almost always	39.71^b^		25.03^d^	
	Always	5.88^a^		7.63^a^	
I generally buy the lowest priced product, regardless of its impact on society/environment	Never	7.35^a^	<0.0001	2.54^a^	<0.0001
	Almost never	23.53^a^		10.58^b^	
	Occasionally	45.59^b^		40.83^c^	
	Almost always	23.53^a^		36.55^c^	
	Always	0.00		9.50^b^	
I strive to avoid products or services that cause environmental damage	Never	0.00	<0.0001	3.48^a^	<0.0001
	Almost never	2.49^a^		15.26^b^	
	Occasionally	54.41^c^		47.66^d^	
	Almost always	33.82^b^		5.62^c^	
	Always	8.82^a^		27.98^a^	

*p-value < 0.05 indicates statistically significant differences in responses between. The same letters in columns indicate homogeneous groups according to Dunn’s procedure.*

The question “Do you think current eating patterns will have adverse effects on the well-being of future generations?” obtained only significant differences at a general level, stating in both surveyed groups that current eating patterns will negatively affect our society in the future (88.2 and 80.6%, respectively, for staff and students).

Regarding the question, “How important is it that your products do not harm the environment?,” “Important” and “very important” were the two more selected categories (83.8 and 73.5%). There were significant differences in age for staff, suggesting that young people (<28 years old) give more importance than people between 44 and 51 because the products they consume do not harm the environment.

The university community generally pointed out that their environmental concerns somewhat affected their shopping habits, being “Affected a little” and “Yes, they are affected” (82.4 and 73.2% staff and students, respectively). Consistently, both groups were surveyed by “Almost always” and “Occasionally” products packaged in reusable containers (83.8 and 80%). Despite obtaining a *p*-value of <0.05 in general, there were only significant differences between gender and age range within the staff. Men of <28 and > 51 and women <28 and 44 to 51 were more likely to report that environmental concerns affected their habits more. Additionally, in both genders, those aged >51 indicated that they bought products packaged in reusable containers.

Regarding the impact of the product price on product choice, both groups nearly half of respondents noted that they occasionally buy cheaper products, even though their environmental impact is more significant (45.6 and 40.8%, respectively, for staff and students) and only occasionally strive to avoid products or services that cause environmental damage (54.4 and 47.7%), a higher percentage of staff versus students searched “always” or “almost always” products and services with reduced environmental impact (42.6% versus 33.7%).

## 4 Discussion

The study at the university, through online surveys, focused on understanding the perceptions of sustainability of students and university staff. They were assessed on their knowledge of the university’s sustainable initiatives, acceptance of the food offered on campus, waste management, and environmental awareness.

The results show that the interviewed population has basic knowledge of sustainability because, in the first question, most respondents of both sexes answered that they knew something about it. Additionally, it was observed that this level of knowledge is accentuated by age and professional maturity. Respondents generally related this concept to renewable energy and with second life and reuse of packaging.

The UPV promotes care for the environment through various initiatives, such as the celebration of the International Day of Food Waste and the agroecological market (that offers seasonal and local food once a week on campus). However, the survey revealed that participation is scarce, especially among young people, often due to unfamiliarity with these activities or lack of interest. Also, there is scarce knowledge of the students at the university units dedicated to sustainability issues. It is important to engage more students with UPV’s environmental initiatives. This information helps the university identify areas for improvement to promote consumption and awareness of eco-friendly alternatives, supporting the university’s green transition ant its daily habits.

This study, in turn, provides information on which social media the different groups considered more effective in achieving a more successful promotion of these initiatives. Additionally, it reveals which barriers they perceive as inhibiting the university from becoming more eco-friendly, and in turn, allows for the development of strategies that focus on them. For example, regardless of their professional profile, most respondents voted that social media platforms like Instagram or LinkedIn and the university’s website or even informational emails were the best option for communicating activities or initiatives. Therefore, the administrative staff’s good management of these services would be crucial to effectively deliver these communications and thus increase participation, which is often scarce in many cases. Additionally, the students noted that a lack of motivation was one of the barriers they encountered, besides a lack of resources, mostly pointed out by the staff. The university must consider both problems to encourage the community toward a greener and more environmentally friendly university. On the other hand, there is a growing concern about people’s health, which has intensified over the years. More and more consumers are looking for natural and healthy foods (De Ridder et al., 2017; Dernini et al., 2017; Oliffe et al., 2017; Vallejo-Alviter and Martínez-Moctezuma, 2017). This survey showed that 72 and 48% of staff and students, respectively, considered they eat a healthy diet based on the Mediterranean diet. Both groups agree that a change in the diet offered at the university is necessary. However, an increase in local or zero-kilometer products does not guarantee a rise in consumption due to the impact on price. The university could promote healthier diets using strategies aimed at increasing nutritional education among the university community, mainly in its cafeterias or vending machines, through posters with nutritional information or healthy tips.

Food waste is a concept well known by participants, and recycling practices are mainly routine inside and outside campus. Through the survey, the participants stated that the university carries out good waste management, although most are unaware of the initiatives associated with this topic. The participants’ recycling practices are primarily routine inside and outside campus, which suggests that the university community is aware of the importance of waste separation in facilitating its management and treatment. A generational gap in the use of services based on the circular economy, such as Too Good to Go, was detected. The use of these platforms is more extended among university students than among staff. As in this study, the average age of the respondents who use this application was between 20 and 34 years, most of whom have a university education. Moreover, it is an appropriate educational tool to reduce food waste and enable the shift toward more sustainable food systems (da Fonte Pacheco, 2022).

Regarding purchasing habits in general, there is a concern about the impact caused by their production, and there is a certain social awareness when buying one product or another. These results agree with previous studies that indicate a growing environmental awareness in academic environments (Morata Verdugo et al., 2020).

The study provides a comprehensive view of perceptions and behaviors related to sustainability within the UPV, identifying strengths (the results highlight a good knowledge base) and areas for improvement, specifically in participation and motivation to maintain healthy and sustainable practices in all areas. Integrating these findings into university policies and programs can facilitate a more effective transition toward sustainable practices and foster an environmentally conscious culture among the entire university community.

## 5 Limitations and future studies

The participation amounted to 2.5% of the students and 2.3% of the staff, representing only some of the university community. According to other authors (Andrade, 2020), the methodological limitations in online surveys could be an inadequate description of the target population and uncontrolled respondents, increasing biases.

For future studies, increasing the number of participants and the duration of data collection is suggested. Mixed methods, including interviews and focus groups, could be used to better understand perceptions and behaviors.

## Data Availability

The datasets utilized in this article are not accessible to readers to safeguard the confidentiality of the individuals interviewed for this research. For more information regarding the interviews conducted, please get in touch with the author of this paper. Requests to obtain the datasets should be addressed to the corresponding author.
